# Editorial: Multiplex Immunohistochemistry/Immunofluorescence Technique: The Potential and Promise for Clinical Application

**DOI:** 10.3389/fmolb.2022.831383

**Published:** 2022-02-25

**Authors:** Rachel Elizabeth Ann Fincham, Hamed Bashiri, Mai Chan Lau, Joe Yeong

**Affiliations:** ^1^ Centre for Tumour Biology, Barts Cancer Institute—A CRUK Centre of Excellence, Queen Mary University of London, London, United Kingdom; ^2^ Institute of Molecular Cell Biology (IMCB), Agency of Science, Technology and Research (A*STAR), Singapore, Singapore; ^3^ Singapore Immunology Network, Agency of Science (SIgN), Technology and Research (A*STAR), Singapore, Singapore; ^4^ Department of Anatomical Pathology, Singapore General Hospital, Singapore, Singapore

**Keywords:** multiplex immunofluorescence, multiplex immunohistochemistry, immunotherapy, spatial technology, spatial biology, digital pathology, clinical translation

Conventional immunohistochemistry (IHC) has long been regarded as the “gold standard” for the diagnosis of tissue pathology. However, the diagnostic-prognostic value of this technique is limited by factors such as high inter-observer variability, restricted labeling potential and insufficient availability of samples for testing (Tan et al., 2020). However, the emergence of multiplex immunohistochemistry/immunofluorescence (mIHC/IF) techniques has provided an opportunity to overcome many of these challenges. These techniques facilitate investigation of multiple biomarkers on a single slide as well as exploration of tissue-level biology, classification of cell-cell interactions, and identification of rare cellular phenotypes. mIHC/IF is also a powerful supplement to technologies such as next generation sequencing. As such, mIHC/IF holds the potential to revolutionize cancer therapies and diagnostic pathology (Tan et al., 2020; Hernandez et al.; Lazcano et al.; Parra). In this special edition of Frontiers in Molecular Biosciences we review the importance and clinical translational potential of mIHC/IF.

The COVID-19 pandemic has resulted in an urgent need to understand the implications of myocardial involvement in disease mortality, necessitating detection of viral components within tissue samples. Chong et al., used a combination of mIHC/IF and molecular techniques to examine cardiac autopsy specimens from 12 intensive care unit (ICU) naïve, SARS-CoV-2 PCR-positive patients. These novel findings revealed histopathologic changes in coronary vessels, as well as inflammation of the myocardium in these patients. This study provided crucial insights into the characteristics of COVID-19 patients at risk of sudden death, and suggested the possibility of long-term complications in patients with persistent virus (Chong et al.).

As well as detecting viral particles within tissue samples, mIHC/IF can be used to explore the tumour immune microenvironment (TIME). Through the development of a multiplex panel for the identification of proliferating B cells, follicular helper T cells, and follicular regulatory T cells, Boisson et al., demonstrated the power of mIHC/IF for studying marker co-localization in individual tissue sections and highlighted the potential application of this technique in clinical practice (Boisson et al.).

To exploit the translational potential of mIHC/IF, Wharton et al., proposed that, until greater levels of standardization across mIHC/IF protocols and pipelines are established, diagnostic laboratories will play a critical role in driving the adoption of multiplex tissue diagnostics through retrospective analysis of clinical trial samples and development of reproducible diagnostic assays (Wharton et al.). In addition, Hoyt provided a discussion of the requirements for the use of mIHC/IF in medical applications and offered suggestions on assay development/improvement. In light of growing health economic concerns in the field of immuno-oncology and the need for tests that precisely predict responses to costly immunotherapies/cell therapies, the translation of mIHC/IF into clinical practice is of paramount importance (Hoyt).

Analysis of the spatial distribution of cells within the tumor microenvironment by mIHC/IF provides important, clinically relevant information; however, with the availability of multiple spatial analysis tools, choosing the correct algorithm remains a challenge. As such, development of robust, standardized analysis pipelines and consensus on their application is necessary to fully exploit the benefits of this technique (Lazcano et al.). In this regard, Parra discussed the analysis of mIHC/IF, with a particular focus on interrogating spatial cellular distribution and concluded that assessment of cell phenotype compartmentalisation and nearest neighbour analysis is the simplest approach to identification of patterns of distribution and cellular interaction (Parra). Similarly, Hernandez et al., noted that in-depth spatial analysis of formalin-fixed, paraffin-embedded patient samples using mIHC/IF facilitates patient stratification for immunotherapy, as well as identification of prognostic and predictive immune biomarkers. Despite these obvious benefits, several limitations were also revealed, including tyramide signal over-reactions, fluorophore constraints and the challenges associated with accurate data interpretation. Thus, a thorough understanding of both the technique and cellular biology are necessary to achieve optimum high-quality data with mIHC/IF (Hernandez et al.).

Apaolaza et al., also highlighted the need for standardized, reproducible image analysis tools for understanding disease pathology and combating the propensity for bias associated with manual analysis. Implementation of such tools may be informative for the improvement and design of novel therapeutic strategies (Apaolaza et al.).

The importance of standardization and quality control is further emphasized by Laberiano-Fernández et al*.* , who highlighted the importance of refining, standardizing and validating the mIHC/IF workflow at the pre-analytical, analytical and post-analytical stages. Laberiano-Fernández et al. also emphasized the importance of antibody selection, optimization and validation as well as the need for the extensive review of panel design and multiplex staining. Similarly, through retrospective assessment, Lazcona et al., demonstrate the importance of assessing tumor content, sample size, and percentages of necrosis and fibrosis for pathology quality control (PQC) in mIHC/IF image analysis (Lazcano et al.). Through workflow standardization and robust PQC, it is hoped that mIHC/IF will become a cornerstone of diagnosis and prognosis in the clinical setting through its incorporation in Clinical Laboratory Improvement Amendments (CLIA) (Laberiano-Fernández et al.).

In conclusion, this special edition encompasses the whole spectrum of current mIHC/IF work, from discovery findings (Boisson et al.; Chong et al.) to translational research (Apaolaza et al.; Hernandez et al.; Hoyt), to guidelines for actual clinical implementation (Laberiano-Fernández et al.; Lazcano et al.). The majority of articles included in this research topic demonstrate the desire of both researchers and clinicians to implement this revolutionary technique into daily clinical practice to ultimately benefit patients. Currently, multiple taskforces and working groups including the Society for Immunotherapy of Cancer (SITC) (Taube et al., 2020) and the Joint Effort to Develop multiplex Immunofluorescence standards (JEDI) council (Nelson et al., 2021; Surace et al., 2021) are working to pave the way for clinical mIHC/IF implementation. Their work includes the writing of expert consensus guidelines and assay checklists; extensive investigation of potential technique errors and generation of technical solutions; standardisation of analysis and evaluation tools; and continued communication with regulatory agents and authorities to understand the gaps and challenges faced in meeting the requirements for clinical application.

With all these efforts, we anticipate that the clinical implementation of mIHC/IF, under proper guidelines and quality assurance/control programs, will come to fruition within the next couple of years.
